# Case report of isolated synchronous multiple splenic metastases from rectal cancer: A case report and brief review of the literature

**DOI:** 10.1097/MD.0000000000029613

**Published:** 2022-08-12

**Authors:** Linxian Zhao, Mingxiu Sui, Jiannan Li, Kai Zhang

**Affiliations:** a Department of General Surgery, The Second Hospital of Jilin University, Changchun, Jilin, China.

**Keywords:** case report, colorectal cancer, isolated splenic metastasis, splenectomy, synchronous

## Abstract

**Introduction:**

Isolated splenic metastasis emanating from colorectal cancer is an extremely rare finding, which usually indicates widely disseminated and multiple metastatic cancer. There have only been 39 cases of isolated splenic metastasis reported in the English literature to date.

**Patient concerns:**

An 84-year-old female patient presented to our department with dark-red bloody stool that had persisted for 1 month and with an increased serum carcinoembryonic antigen (CEA) level.

**Diagnoses:**

A colonoscopy showed a rectal mass located 3 cm from the anal margin, which was 45 mm in diameter. The patient was diagnosed with rectal cancer with splenic metastases by abdomen computed tomography.

**Interventions:**

The patient underwent a radical resection of rectal cancer and splenectomy, and the postoperative histopathology confirmed that the splenic lesions were derived from the adenocarcinoma of the rectum.

**Outcomes:**

After surgical treatment, the patient recovered well and was recommended for further chemotherapy.

**Conclusions:**

In addition to revealing a rare case, we also performed a literature review, including a brief discussion about the atypical isolated splenic metastasis from colorectal cancer. Our findings enrich the database of this rare clinical entity and provide experience in the management of splenic metastasis.

## 1. Introduction

Colorectal cancer (CRC) is the third most common cancer worldwide and the fourth leading cause of death.^[1,2]^ In particular, CRC-related mortality in elderly patients is very high, even after radical surgical resection and new chemotherapy treatments.^[2]^ During clinical examination and diagnosis, most patients with CRC present tumor metastasis to regional lymph nodes, liver, lung, bone, and brain.^[2,3]^ However, both the primary and metastatic tumors of the spleen are exceedingly rare because the spleen consists of a mass of lymphoid tissue.^[4]^ More specifically, the rarity of splenic metastasis can be explained by the special anatomical structure and immunological features of the spleen.^[5]^ Splenic metastasis has been discovered from multiple primary tumors, such as liver cancer, breast cancer, lung cancer, ovarian cancer, and melanoma.^[6,7]^ In this context, isolated splenic metastasis derived from colorectal carcinoma is even rarer and only a few cases have been documented in the literature.^[8]^ Based on the formation time of metastasis, splenic metastasis can be divided into synchronous and metachronous metastasis. Synchronous metastasis means that the splenic lesion is discovered by imaging studies together with the primary tumor. Differently, the diagnosis of metachronous metastasis is usually made during the follow-up of patients in the postsurgical period. Most patients with splenic metastasis are asymptomatic, and only a few cases have presented with spontaneous rupture of the spleen and splenic abscess.^[9]^ For example, 1 patient presented with abdominal pain, hyperpyrexia (40°C), shaking, and chills. Further clinical examination discovered synchronous isolated splenic metastasis and a concomitant splenic abscess.^[10]^ In the present study, we reported a case of synchronous splenic metastasis in an 84-year-old female patient, which was derived from a moderately differentiated adenocarcinoma of the rectum.

## 2. Case report

An 84-year-old female patient came to our department with dark-red bloody stool that has persisted for 1 month. The patient also reported abdominal pain and abdominal distension. She had no symptoms of vomiting, diarrhea, fever, or weight loss, and there was no splenomegaly and hypersplenism. Her blood pressure was 125/60 mm Hg and the pulse rate was 56/min, and other physical examinations were normal. She denied any history of cancer, hypertension, and diabetes. She had a surgery history of cholecystectomy 2 years ago in our hospital. This study was approved by the ethics committee of the Second Hospital of Jilin university.

**Figure 1.**
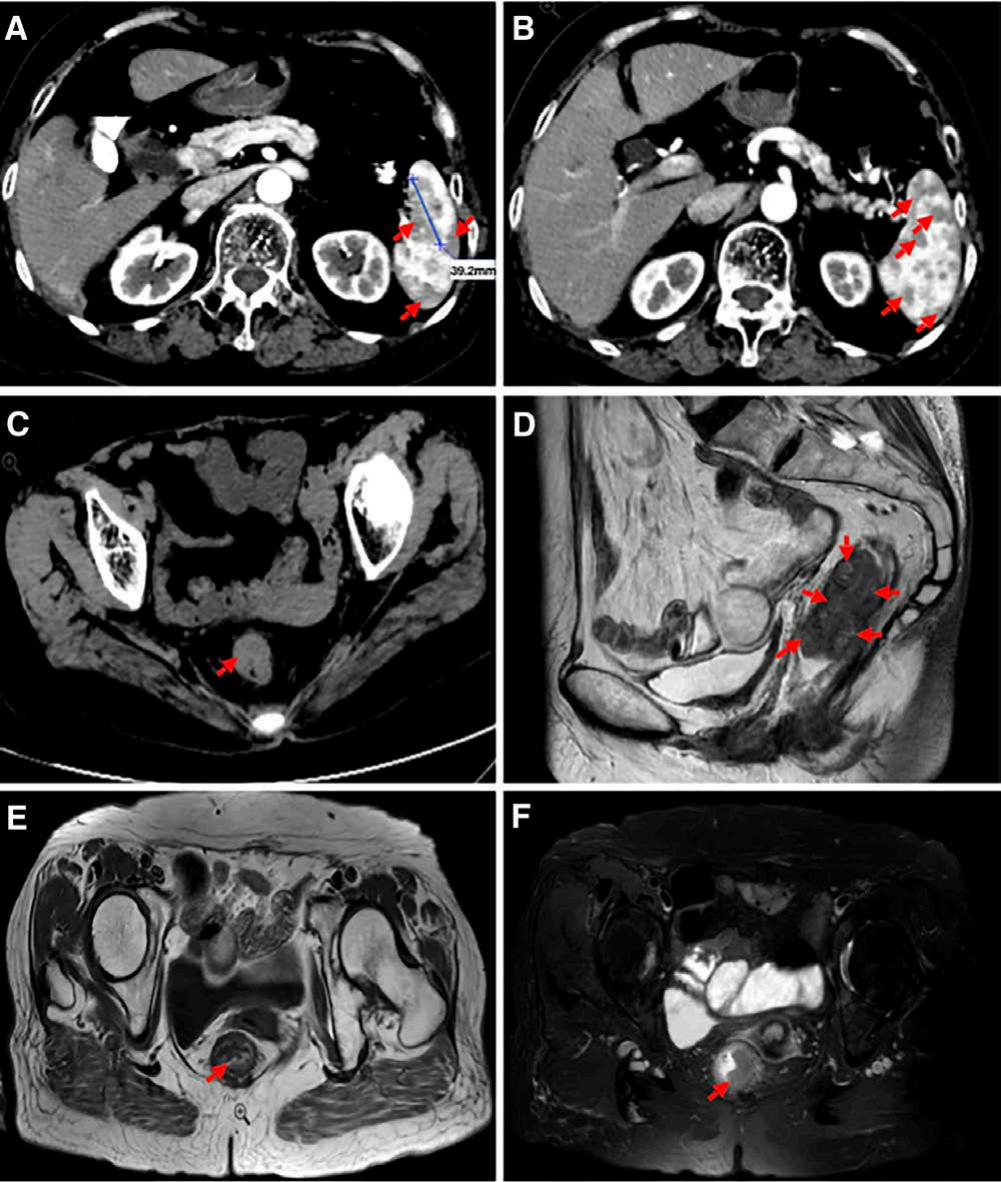
Imaging examinations of the spleen and the rectum. (A and B) Abdomen enhanced CT showing multiple low-density shadows in the spleen (red arrow), the largest diameter of which is 39.2 mm (blue lines). (C) Abdomen CT showing wall thickening of the upper rectum (red arrow). (D) MRI (T2W-TSE-HR) showing that the cumulative length of the tumor was about 57 mm, the lower margin of the tumor was higher than the rectal ring, and the distance from the anorectal ring was about 20 mm (red arrow). (E) MRI (TSE-axial) showing irregular wall thickening of the middle and upper rectum, and the rectal lesion is about 59 mm from the anal margin (red arrow). (F) MRI (T2W-SPAIR-tra) showing a slightly high signal of the rectal lesion (red arrow). CT = computed tomography, MRI = magnetic resonance imaging.

The preoperative laboratory examinations demonstrated total white blood cell count of 2.7 × 10^9^/L (normal: (3.5–9.5) × 10^9^/L) and total protein of 58.1 g/L (normal: 65–85 g/L). The serum levels of alpha-fetoprotein and carbohydrate antigen 19-9 (CA19-9) were within normal limits, except for carcinoembryonic antigen (CEA), which was 57.57 ng/mL (normal 0–3 ng/mL). A colonoscopy showed a rectal mass located 3 cm from the anal margin, which was 45 mm in diameter, with surface depression, erosion, and a propensity for bleeding. The tumor prevented endoscope insertion for further examination of the intestines. The histopathological results revealed that the tumor was a moderately differentiated adenocarcinoma. The abdomen enhanced computed tomography revealed wall thickening of the upper rectum (Fig. 1C) and multiple low-density shadows in the spleen (Fig. 1B), among which the largest diameter was 39.2 mm (Fig. 1A). The radiologist suspected a rectal tumor with splenic metastasis. Magnetic resonance imaging (MRI) showed irregular wall thickening of the middle and upper rectum, and the rectal lesion was about 59 mm from the anal margin. The clinical stage assessed by MRI before surgery was cT3N1 (Fig. 1D, E, and F).

**Figure 2.**
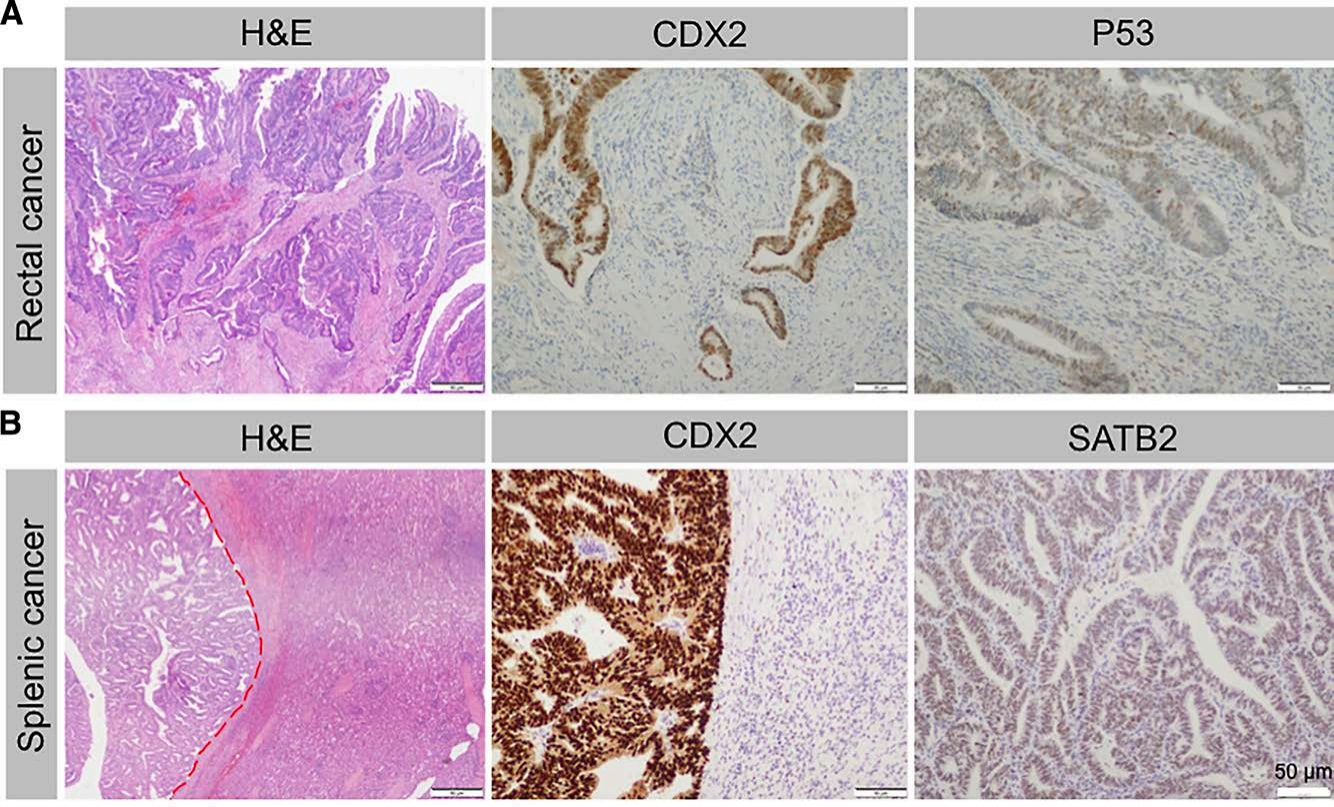
Histological findings of (A) the primary rectal cancer and (B) splenic metastasis. (A) Moderately differentiated adenocarcinoma (HE, ×50); CDX2 positively expressed (CDX2, ×50); *P53* gene mutation positive (P53, ×50). (B) Splenic tumor showing glandular pattern consistent with metastasis from rectal cancer (HE, ×50); CDX2 positively expressed (CDX2, ×50); SATB2 positively stained (SATB2, ×50). HE = hematoxylin and eosin, SATB2 = AT-rich sequence-binding protein 2.

Subsequently, the patient underwent a radical resection for rectal cancer and splenectomy after a series of preoperative examinations. Intraoperatively, we did not find any metastasis and dissemination to other organs. Therefore, a subtotal proctectomy with side-to-side sigmoid colon–rectum anastomosis was performed. The postoperative histopathological results confirmed that the splenic lesions were consistent with adenocarcinoma of the rectum (Fig. 2), which supported the diagnosis of isolated splenic metastasis.

Intraoperatively, seventeen lymph nodes were removed, and postoperative pathology examination did not detect lymph node metastasis. The pathological stage was pT3N0M1 (stage IV). By the 15th postoperative day, the CEA level dropped to 10.13 µg/L, and the patient was finally discharged. The patient was recommended for further chemotherapy and postoperative recovery after surgery was uneventful.

## 3. Discussion

Tumor metastasis is a complex process, which can be influenced by multiple factors, such as anatomical structures, mechanical factors, immunological tissue microenvironment, and intrinsic characteristics of tumor cells.^[11]^ Compared with the liver, lung, and kidney, metastatic tumors of the spleen are difficult to implant, which might be caused by its specific anatomical factors and immune surveillance functions. Especially for rectal cancer, it is difficult to form a splenic metastasis by blood vessel invasion. On the one hand, the venous blood flow above the dentate line can directly flow into the splenic vein through the inferior mesenteric artery and then enter the portal vein system. Thus, the blood flow is from the spleen to the liver, and the retrograde venous blood from the portal vein system to the spleen is very rare, making it difficult for tumor cells to reach the spleen. On the other hand, the venous blood flow below the dentate line can flow into the inferior vena cava through the internal iliac vein and internal pudendal vein. Thus, it is almost impossible to directly enter the vasculature of the spleen. In addition, the acute angulations of the spleen artery and the rhythmic contraction of the splenic capsule significantly limit the ability of the tumor embolus to implant in the spleen.^[12]^ Besides, the reticuloendothelial system of the spleen is capable of inhibiting tumor cell proliferation. Furthermore, the lack of afferent lymphatics also limits lymphogenic metastases. More interestingly, some researchers proposed that splenic cells possess a phagocytic capability and can produce multiple antitumor substances, which can effectively inhibit the progression of tumors.^[13]^ Significantly, 1 study also proposed that although disseminated cancer cells can easily reside in splenic parenchyma, the special microenvironment of the spleen may suppress the growth and progression of these cells.^[4]^ Consequently, the splenic micrometastatic foci cannot be detected through traditional clinical methods, resulting in the clinically detectable isolated metastases of the spleen being reported as 4.4% for colon cancer and 1.6% for rectum cancer.^[14]^ In comparison, the incidence of splenic micrometastases at autopsy is approximately 7.1%.^[15]^

Splenic metastasis has to be distinguished from the primary splenic lesion, such as malignant lymphoma, vascular tumors, infections disease, septic emboli, and granulomatous diseases.^[4]^ Recently, with the development of medical imaging techniques, such as positron emission tomography (PET)-CT and PET-MRI, it has become easier to detect splenic metastases, thereby increasing their apparent incidence. Consequently, it is important to trace the patient’s disease history, in which a history of malignancy increases the possibility of splenic metastasis.^[16]^ Interestingly, 1 study reported a patient with rectosigmoid adenocarcinoma with splenic lesions; however, the postoperative pathological diagnosis revealed a primary splenic malignant lymphoma.^[17]^ Therefore, histopathology remains the gold standard for diagnosis.

In this study, we analyzed 39 previously published cases (22 males, 17 females; age range, 33 to 84 years; mean, 64 years) of isolated splenic metastasis derived from CRC (Table 1).^[5,8,10,14,18–32,34–46]^ Among them, 35 cases were metachronous metastasis and only 4 cases were synchronous metastasis. Interestingly, in terms of splenic lesions, there were only 4 cases of multiple metachronous splenic metastases, and most cases^[34]^ were solitary. Here, we described the first case of a synchronic splenic metastasis from a malignant tumor of the rectum.

**Table 1:** Isolate splenic tumor metastases derived from CRC.

**No.**	**Age/sex**	**Primary tumor site**	**Stage**	**Size (cm**)	**Synchronous/metachronous**	**DFI**	**Solitary/multiple**	**CEA** **ng/mL**	**Imaging**	**Treatment**	**The prognosis**	**Ref**
1	48/F	Sigmoid	III	0.4–3	Metachronous	21 mo	Multiple	206.8	PET and MRI	S, Cmt, TT	7 mo, alive	^[18]^
2	73/M	Hepatic flexure	IV	5.7	Synchronous	NA	Solitary	6.9	CT	S, Cmt	6 mo, alive	^[19]^
3	76/F	Descending	III	1.6	Metachronous	28 mo	Solitary	NA	PET	S, Cmt	21 mo, alive	^[7]^
4	84/F	Cecum	III	8	Metachronous	5 mo	Solitary	205	CT and MRI	S	NA	^[8]^
5	53/M	Sigmoid	NA	NA	Metachronous	12 mo	Solitary	NA	PET	S, Cmt	36 mo, died	^[20]^
6	59/M	Descending, sigmoid	NA	NA	Metachronous	3 mo	Solitary	NA	PET	S, Cmt	12 mo, alive	^[20]^
7	64/F	Cecum	I	4.9	Metachronous	6 mo	Solitary	38	CT	S, Cmt	10 mo, alive	^[21]^
8	62/F	Sigmoid	III	3–6	Metachronous	36 mo	Multiple	NA	CT	S. Cmt	10 mo, alive	^[22]^
9	74/M	Descending	IV	7.5–8.5	Synchronous	NA	Multiple	242	CT	S, Cmt	NA	^[2]^
10	74/M	Cecum	III	7	Metachronous	36 mo	Solitary	38.6	PET	S, Cmt	12 mo, alive	^[23]^
11	58/M	Cecum	III	3.5–5.5	Metachronous	20 mo	Solitary	4.62	PET	Cmt	7 mo, alive	^[14]^
12	70/M	Splenic flexure	III	10	Metachronous	24 mo	Solitary	NA	CT	S, Cmt	12 mo, alive	^[24]^
13	73/M	Hepatic flexure	III	1,5	Metachronous	62 mo	Solitary	132	CT-PET	S, Cmt	36 mo, alive	^[25]^
14	52/F	Sigmoid	III	4.5	Metachronous	37 mo	Solitary	16	PET	S, Cmt	NA	^[26]^
15	69/F	Sigmoid	II	4	Metachronous	24 mo	Solitary	20	CT	S, Cmt	60 mo, alive	^[27]^
16	80/F	Transverse	III	8	Metachronous	9 mo	Solitary	52.3	NA	S	NA	^[28]^
17	54/F	Splenic flexure	III	4,5	Synchronous	NA	Multiple	31.1	CT	S, Cmt	NA	^[10]^
18	52/M	Sigmoid, rectum	II	13	Metachronous	72 mo	Solitary	7.2	CT	S, Cmt	22 mo, alive	^[29]^
19	76/M	Splenic flexure	III	6.5	Metachronous	14 mo	Solitary	95	CT and PET	S	12 mo, alive	^[30]^
20	52/F	Sigmoid	NA	NA	Metachronous	24 mo	Solitary	Rise	CT	S	22 mo, died	^[31]^
21	62/M	Sigmoid	II	3	Metachronous	25 mo	Solitary	NA	CT	S	21 mo, alive	^[31]^
22	65/M	Ascending	II	5	Metachronous	36 mo	Solitary	10.9	CT	S	18 mo, alive	^[32]^
23	78/F	Rectum	III	18	Metachronous	48 mo	Solitary	64	CT	S	84 mo, alive	^[5]^
24	72/M	Sigmoid	III	9	Metachronous	48 mo	Solitary	106	LSS	S	6 mo, alive	^[33]^
25	81/M	Cecum	III	NA	Metachronous	30 mo	Solitary	7.5	LSS	S	12 mo, alive	^[34]^
26	51/F	Rectum	II	2.8	Metachronous	51 mo	Solitary	13.5	CT	S	14 mo, alive	^[35]^
27	72/F	Descending	II	3	Metachronous	144 mo	Solitary	223	CT	S	12 mo, alive	^[36]^
28	62/F	Descending	III	4	Metachronous	42 mo	Solitary	Rise	CT	S	12 mo, alive	^[37]^
29	74/M	Sigmoid	II	9.5	Metachronous	24 mo	Solitary	23.4	CT	S	24 mo, alive	^[38]^
30	52/M	Ascending	NA	NA	Metachronous	12 mo	Solitary	NA	US and CT	S	6 mo, alive	^[39]^
31	48/M	Ascending	NA	NA	Metachronous	24 mo	Solitary	NA	US and CT	S	3 mo, alive	^[39]^
32	33/F	Sigmoid	III	3.5	Metachronous	3 mo	Solitary	9	CT and MRI	S	12 mo, alive	^[40]^
33	51/M	Sigmoid	III	13	Metachronous	72 mo	Solitary	NA	CT	S	6 mo, alive	^[41]^
34	72/M	Rectum	III	NA	Metachronous	18 mo	Solitary	Rise	CT	S	NA	^[42]^
35	59/M	Ascending	III	4	Metachronous	15 mo	Solitary	37	CT	S	24 mo, alive	^[43]^
36	78/M	Cecum	III	7	Metachronous	37 mo	Solitary	38.6	CT and PET	S, Cmt	9 mo, alive	^[23]^
37	76/F	Descending	III	1.6	Metachronous	24 mo	Solitary	NA	PET	S, Cmt	21 mo, alive	^[44]^
38	62/M	Sigmoid	III	3.5	Metachronous	23 mo	Solitary	2.5	US and CT	S	19 mo, alive	^[45]^
39	52/F	Descending	Ⅳ	5	Synchronous	NA	Solitary	Rise	CT	S	12 mo, died	^[46]^

Among these cases, based on their primary tumor sites, we observed that the most common lesion was in the sigmoid colon (12 cases), accounting for 30.8%. In comparison, the 2 most uncommon sites were the transverse colon in 1 case and the hepatic flexure in 2 cases. Others included 6 cases in the cecum, 4 cases in the ascending colon, 3 cases in the splenic curvature, 6 cases in the descending colon, and 3 cases in the rectum. In particular, 2 patients presented with multiple primary cancers. For the 1 patient, the primary tumors were found in the descending colon and sigmoid colon together, and the tumors of the other patient were located in the sigmoid colon and rectum. Based on these findings, we deduced that the primary tumors of splenic metastasis are most commonly found in the left hemicolon in 24 cases, accounting for 61.5%, which might reflect the fact that these tumor cells can enter counter-currently into the splenic vein via the inferior mesenteric vein. In terms of primary tumor stage (1 case with stage I, 7 cases with stage II, 23 cases with stage III, 3 cases with stage IV, and 5 cases in which the stage was not mentioned), we discovered that most isolated splenic metastasis is derived from the median or advanced CRC.

Among reported cases, 29 of the 39 patients presented with an elevated CEA level. In accordance with the above results, the CEA level reached a maximum of 57.57 ng/mL in our case. In terms of cases of metachronous splenic metastasis cases, the disease-free interval ranged from 3 to 144 months (average, 31.7 months). In addition, for most of the patients, the isolated splenic metastases were found during postoperative follow-up by radiological examinations, such as abdomen computed tomography, MRI, ultrasound, fine-needle aspiration, and even fluorodeoxyglucose-PET. According to the literature, only 1 patient did not undergo curative splenectomy. For most patients, splenectomy and chemotherapy were the 2 main optimal treatment strategies.

In our case, both the primary rectal cancer and metastasis splenic lesions showed caudal type homeobox 2 (CDX2) expression (Fig. 2). CDX2, a homeobox protein, is believed to be an important factor in maintaining the intestinal phenotype and regulating colorectal tumor metastasis.^[47]^ Importantly, we observed that splenic lesions express special AT-rich sequence-binding protein 2 (Fig. 2), which is used as a diagnostic marker of colorectal origin cancer.^[48]^ One study found that >93% of colorectal origin tumors showed AT-rich sequence-binding protein 2 positive staining, which was consistent with our results.^[49]^

## 4. Conclusion

We reported a case of the rare occurrence of isolated synchronous multiple splenic metastases from rectal cancer. Our findings enrich the database of this rare clinical entity and provide experience in the management of splenic metastasis. To the best of our knowledge, splenic metastasis of colorectal carcinoma is very uncommon. With improvements in examining techniques, increasing numbers of patients with isolated splenic metastasis might be found. Over the long term, it is essential to follow-up patients with CRC postoperatively, which could effectively improve the management and prolong the survival of these patients with isolated splenic metastases. The common therapeutics options include splenectomy, chemotherapy, targeted therapy, and radiotherapy. However, to date, only a small number of cases have been reported and long-term follow-up is absent. Therefore, standardized clinical treatment strategies for splenic metastasis have not been established. In future studies, more attention should be paid to this rare entity.

## Author contributions

Conceptualization: Linxian Zhao, Mingxiu Sui

Investigation: Kai Zhang, Jiannan Li

Methodology: Kai Zhang, Mingxiu Sui

Writing – original draft: Linxian Zhao

Writing – review & editing: Kai Zhang, Jiannan Li
